# Comparative Lipidomics and Proteomics of Lipid Droplets in the Mesocarp and Seed Tissues of Chinese Tallow (*Triadica sebifera*)

**DOI:** 10.3389/fpls.2017.01339

**Published:** 2017-08-02

**Authors:** Yao Zhi, Matthew C. Taylor, Peter M. Campbell, Andrew C. Warden, Pushkar Shrestha, Anna El Tahchy, Vivien Rolland, Thomas Vanhercke, James R. Petrie, Rosemary G. White, Wenli Chen, Surinder P. Singh, Qing Liu

**Affiliations:** ^1^State Key Laboratory of Agricultural Microbiology, Huazhong Agricultural University Wuhan, China; ^2^CSIRO Agriculture and Food Canberra, ACT, Australia; ^3^CSIRO Land and Water Canberra, ACT, Australia

**Keywords:** lipidomics, proteomics, lipid droplets, Chinese tallow, *Triadica sebifera*, lipid biosynthesis, oleosin, LDAP

## Abstract

Lipid droplets (LDs) are composed of a monolayer of phospholipids (PLs), surrounding a core of non-polar lipids that consist mostly of triacylglycerols (TAGs) and to a lesser extent diacylglycerols. In this study, lipidome analysis illustrated striking differences in non-polar lipids and PL species between LDs derived from *Triadica sebifera* seed kernels and mesocarp. In mesocarp LDs, the most abundant species of TAG contained one C18:1 and two C16:0 and fatty acids, while TAGs containing three C18 fatty acids with higher level of unsaturation were dominant in the seed kernel LDs. This reflects the distinct differences in fatty acid composition of mesocarp (palmitate-rich) and seed-derived oil (α-linoleneate-rich) in *T. sebifera*. Major PLs in seed LDs were found to be rich in polyunsaturated fatty acids, in contrast to those with relatively shorter carbon chain and lower level of unsaturation in mesocarp LDs. The LD proteome analysis in *T. sebifera* identified 207 proteins from mesocarp, and 54 proteins from seed kernel, which belong to various functional classes including lipid metabolism, transcription and translation, trafficking and transport, cytoskeleton, chaperones, and signal transduction. Oleosin and lipid droplets associated proteins (LDAP) were found to be the predominant proteins associated with LDs in seed and mesocarp tissues, respectively. We also show that LDs appear to be in close proximity to a number of organelles including the endoplasmic reticulum, mitochondria, peroxisomes, and Golgi apparatus. This comparative study between seed and mesocarp LDs may shed some light on the structure of plant LDs and improve our understanding of their functionality and cellular metabolic networks in oleaginous plant tissues.

## Introduction

Plants accumulate oil or non-polar lipids mainly as triacylglycerols (TAGs) which are needed for seed development and energy mobilization during seed germination and early seedling establishment prior to photosynthetic autotrophy (Huang, 1996; Lung and Weselake, 2006). TAGs generally accumulate poorly in non-seed tissues, except in the mesocarp tissues from a few woody plants, such as oil palm (*Elaeis guineensis*), olive (*Olea europaea*), avocado (*Persea americana*), Chinese tallow (*Triadica sebifera*), and the stalon tuber of nutsedge (*Cyperus esculentus*), as reviewed recently by Rahman et al. (2016).

TAGs are synthesized in distinct regions of the endoplasmic reticulum (ER) and are sequestered between the two leaflets of the ER membrane along with corresponding enzymes and structural proteins and budded off as oil bodies or lipid droplets (LD) when their size reaches a certain threshold (Chapman et al., 2012; Laibach et al., 2015). LDs have a single-layer phospholipid (PL) membrane with the polar head groups in contact with the cytosol and the non-polar tails in contact with the internal TAG. Until recently, plant LDs have been largely regarded as merely static cytosolic TAG inclusions, but there is increasing evidence demonstrating that LDs are complex and dynamic organelles with a crucial role in various metabolic activities (Ducharme and Bickel, 2008; Chapman et al., 2012).

Compared to other cellular organelles such as ER and mitochondria, LDs are poorly understood. They consist of only two components, lipids and proteins, and studies of these are of critical importance for understanding the biogenesis, mobilization, and functionality of LDs. LDs were first isolated and analyzed from yeast and mammal tissues and cells (Nissen and Bojesen, 1969; Hood and Patton, 1973; Comai et al., 1975), but studies on plant LD are relatively recent and information on both lipid and protein compositions of plant LD is still rather limited. As a main source of vegetable oil, seed LDs have been studied in a few important oilseed crops, including rapeseed (*Brassica napus*) (Katavic et al., 2006; Jolivet et al., 2009), Jatropha (*Jatropha curcas*) (Popluechai et al., 2011; Liu H. et al., 2015), maize (*Zea mays*) (Tnani et al., 2011), and sunflower (*Helianthus annuus*) (Thakur and Bhatla, 2015). In contrast, relatively little attention has been paid to LDs from other plant tissues despite their presence in most cells (Laibach et al., 2015). To our knowledge, proteomic analysis of LDs in non-seed plant tissues has so far been only reported for *P. americana* mesocarp (Horn et al., 2013).

*T. sebifera*, a rapidly growing and deciduous tree from the family Euphorbiacae, is attracting increasing attention as a novel source of vegetable oil (Jeffrey and Padley, 1991). The seed kernel which is mainly endosperm, consists of ~40–50% oil, referred to as “stillingia,” which can be used as a drying oil in formulating paints and varnishes (Bolley and McCormack, 1950). The waxy layer outside the seed coat, derived from mesocarp tissue, is also rich in oil that is used in the production of soap, cosmetics, candles, or as a cocoa butter substitute (Heywood, 1993; Facciola, 1999). It has been estimated that yields of *T. sebifera* oil derived from both seed and mesocarp could reach 4,700 L ha^−1^ (Shupe and Catallo, 2006). Despite the current hurdle for large scale cultivation of *T. sebifera* in many parts of the world due to its invasiveness (Jubinsky and Anderson, 1996), there is a considerable potential for adaptation of such a high-yielding oil plant species for biodiesel production and the oleochemical industry (Shupe and Catallo, 2006).

The fatty acid composition of seed and mesocarp oils derived from *T. sebifera* has been reported (Yang et al., 2012). Further in-depth studies aimed at determining the LD lipid composition and the ratio of TAG vs. membrane PL, and identifying proteins required for the stability and activity of LD, would facilitate a better understanding of the molecular and biochemical mechanism of LD formation and dynamics, and allow the development of tailored LDs in both seed and vegetative tissues.

Proteomic studies on plants focusing on the seed LDs have so far revealed mainly oleosin and to a lesser extent caleosin as the two major LD proteins that play a crucial role in determining the size of LDs, maintaining LD structural stability and withstanding the strains of both dehydration and rehydration in seeds (Siloto et al., 2008; Chapman et al., 2012). Transcriptomic and proteomic studies have revealed that *OLEOSINs* are poorly expressed in non-seed oleaginous mesocarp tissues of *E. guineensis* and *P. americana* (Horn et al., 2013; Loei et al., 2013; Kilaru et al., 2015). Instead, a small family of LD-associated proteins (LDAPs) belonging to the rubber elongation factor (REF) superfamily have been identified as the main proteins associated with the surface of mesocarp LDs where they play a key role in maintenance of structural integrity while allowing dynamic functions (Kim et al., 2010, 2016; Gidda et al., 2013, 2016; Horn et al., 2013).

In this paper, we focus on the cellular localization of LDs, and compositional analyses of lipids and proteins associated with LDs in *T. sebifera* seed and mesocarp tissues. Lipidomic analysis or the profiling of lipid fractions was carried out using liquid chromatography-mass spectrometry (LC-MS) to obtain a detailed insight into the polar and non-polar lipid species present in LDs. Comparative proteomic analysis between seed and mesocarp LDs may bridge some of the fundamental gaps in knowledge by significantly addressing the limited understanding of compositions of lipids and proteins in LDs. In addition, our results may provide insights into LD formation, lipid metabolism and how LDs might be connected with multiple metabolic networks, especially in the rather poorly studied but potentially important oil-producing plant, *T. sebifera*. Finally, investigations into molecular and cellular biological aspects of LDs enabled the development of an integrated picture of plant LDs, which would be useful for future research in this area.

## Materials and methods

### Plant materials

**Figure 1:**
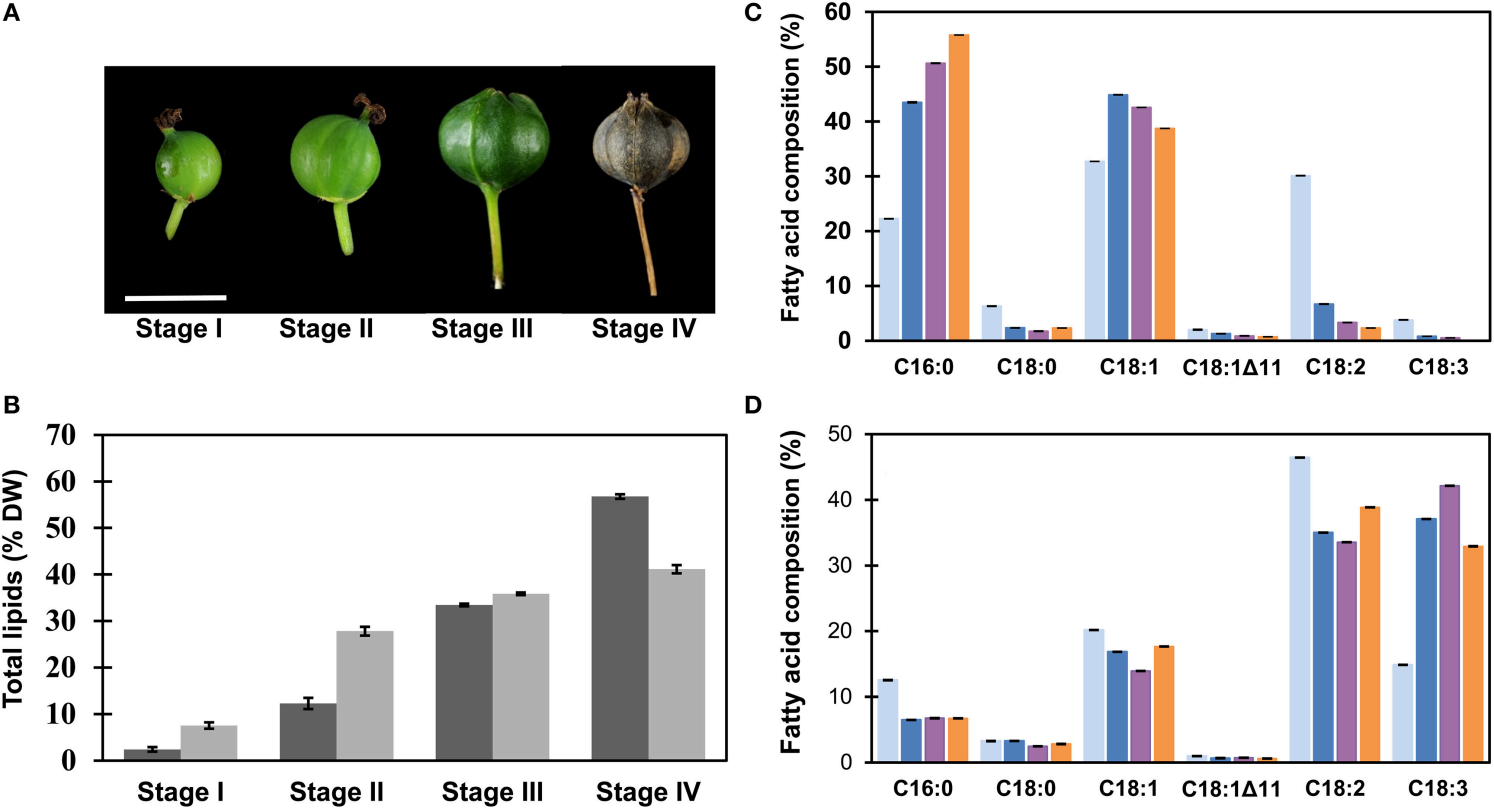
Analysis of total lipids during fruit development of *Triadica sebifera*. **(A)** Sampling stages. Fruit development period was divided into four stages, including stage I (58 DAA), II (76 DAA), III (91 DAA), and IV (120 DAA). Scale bar = 10 mm. **(B)** The contents of total lipids in mesocarp (dark) and seed kernel (shade) tissues in *T. sebifera* during fruit development on the basis of dry weight (DW). **(C)** Fatty acid composition of the total lipids isolated from developing *T. sebifera* mesocarp during fruit development. Stage I (gray), stage II (blue), stage III (purple), and stage IV (orange). **(D)** Fatty acid composition of the total lipids isolated from developing *T. sebifera* seed kernels. Stage I (gray), stage II (blue), stage III (purple), and stage IV (orange). Mean ± Standard deviation (*n* = 3).

From the onset of flowering, fruits were tagged on the *T. sebifera* trees grown on the Australian National University campus in Acton, Canberra, Australia (35.2777° S, 149.1185° E). The estimated fruit development stage is presented as days after anthesis (DAA). For the sake of simplicity, we have classified the fruit development into four stages (I–IV) as illustrated in Figure 1A. Stage I (58 DAA) is characterized by the initiation of seed embryo formation and fruit development. At this stage, the fruit was the size of a typical match head and yellowish-green in color. At stage II (76 DAA), inside a fruit, the seed coat started to become woody and brown, while the color of the mesocarp turned from creamy-green to white as the fruit size expanded to its maximum. Fruits at the stage III (91 DAA) exhibited a clearly defined trilobular shape and dark-green fruit coat. At stage IV (120 DAA), the trilobular fruit cracked open, and mesocarp tissue was seen as a thin, waxy layer outside the seed coat.

### Lipid analysis

The total lipids from developing mesocarp and seed kernel were extracted and fatty acid methyl esters (FAMEs) were prepared as previously described (Liu et al., 2017). FAMEs were analyzed by gas chromatograph (GC) 7890A (Agilent Technologies, CA) equipped with a 30 m BPX70 column (SGE, Austin, TX). Peaks were integrated with ChemStation software Rev B.04.03 (Agilent Technologies).

### Isolation of LD

**Figure 2:**
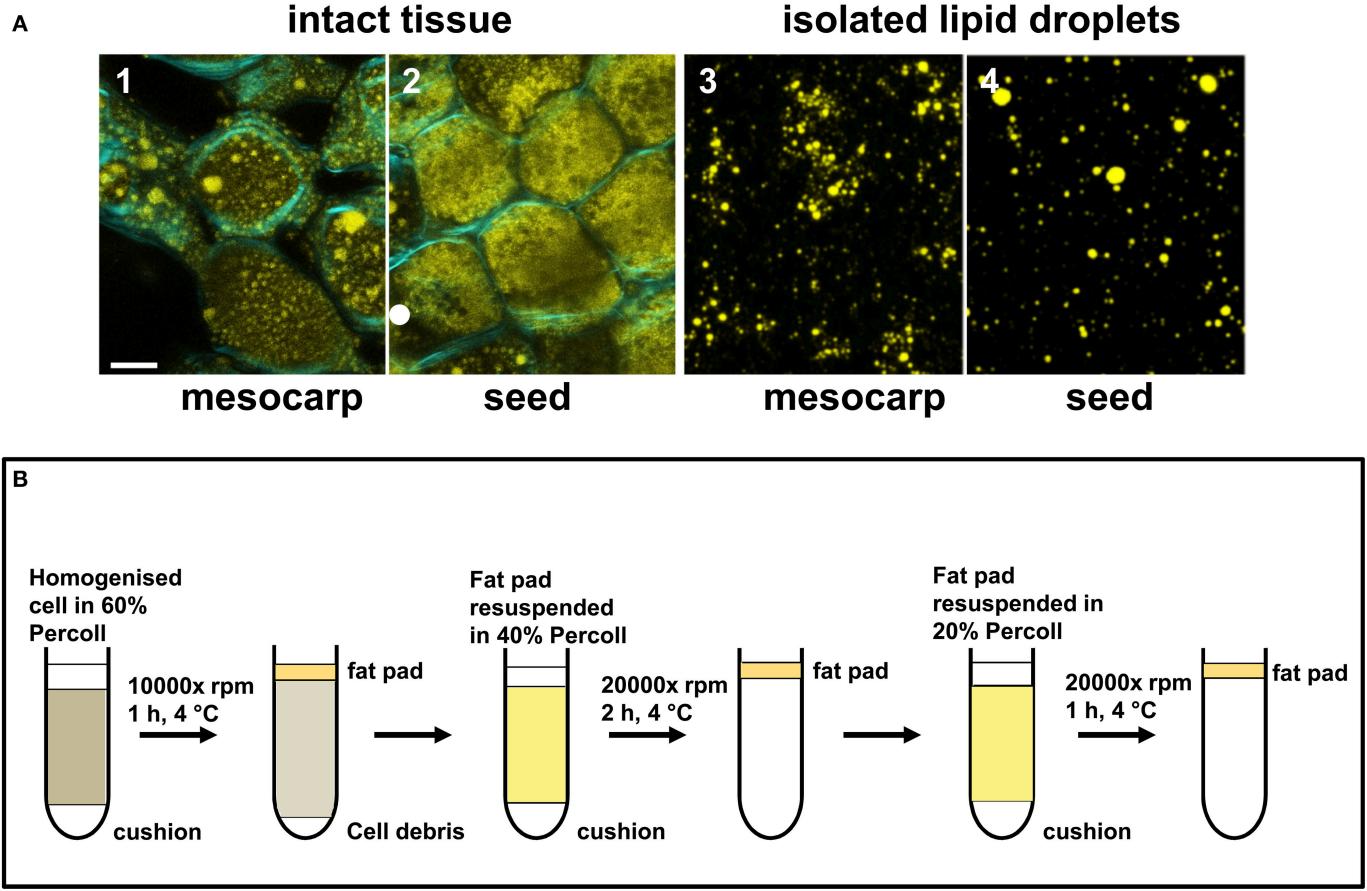
Isolation of LDs from *Triadica sebifera* mesocarp and seed kernel. **(A)** Confocal images showing the presence of LDs in the intact tissues of mesocarp (1) and seed kernel (2) at the stage III of fruit development (Scale bar = 10 μm). The “fat pads” isolated from *T. sebifera* mesocarp (3) and seed kernel (4) tissues were stained by Nile Red, which illustrates the presence of intact LDs (Scale bar = 10 μm). **(B)** A schematic summary of the procedures used for the isolation and purification of LDs.

*T. sebifera* fruits were harvested at 91 DAA. Approximately 3.5 g of mesocarp tissue and 1.5 g of seed kernel tissue were excised on ice and homogenized with an IKA Disperser T10 basic homogenizer in a homogenization solution containing 100 mM potassium phosphate buffer (pH7.2), 1 mM EDTA, and 100 mM KCl with 60% Percoll (Sigma-Aldrich, MO, USA). After being filtered through three layer of Miracloth, the cell extracts were subjected to Percoll gradient centrifugation in a Beckman L8-70M centrifuge with a swinging bucket rotor at 10,000x rpm for 1 h at 4°C (Figure 2B). A cushion of 60% Percoll in homogenization solution was laid at the bottom of the tube. Four volumes of cell extract, suspended in homogenization solution with 60% Percoll was placed on the top of the cushion. Following centrifugation, the top “fat pad” layer formed was removed using a stainless steel spatula and a Pasteur pipette for further purification. LDs were purified by two additional series of Percoll gradient centrifugation with decreased Percoll concentrations at 40 and 20%, respectively, each at 20,000x rpm for 1 h at 4°C. The floating “fat pads” containing purified LDs were collected and examined under confocal microscope for their purity and integrity before they were resuspended into 100 μL homogenization buffer. Four hundred microliters of Methanol, 100 μL chloroform and 300 μL H_2_O were added to the sample and mixed thoroughly by vortex prior to phase separation at 16,163x g with a benchtop centrifuge. The lower chloroform phase was removed for lipidomics analysis. Methanol (300 μL) was added to the upper, aqueous phase, mixed vigorously, and centrifuged (16,163x g) to precipitate protein.

### Liquid chromatography-mass spectrometry of isolated LD proteins

The precipitated protein was dissolved by grinding and heating in SDS-PAGE sample buffer. The proteins were partly fractionated and separated from any residual Percoll particles by running the tracking dye approximately 1 cm into a pre-cast polyacrylamide gel (4–12% Bis-Tris, NuPAGE, Thermo Fisher Scientific, Waltham, MA). The region of the gel showing Coomassie-stained proteins (Aquastain, Geneworks, Thebarton, SA, Australia) was isolated and diced into ~1 mm cubes for in-gel digestions with trypsin and liquid chromatography-tandem mass spectral analysis (LC-MS) as previously described using an Agilent Chip Cube system coupled to an Agilent Q-TOF 6550 mass spectrometer (Campbell et al., 2014).

Each in-gel digestion was analyzed by LC-MS and repeated with four different sample loadings (1, 2, 4, and 8 μL). The complete set of mass spectra from each “fat pad” preparation was used to search a database containing all available predicted protein sequences from the *T. sebifera* fruit transcriptome database (Divi et al., 2016). The search was performed with SpectrumMill software (Agilent Rev B.04.01.141 SP1) and the following settings: precursor mass tolerance of 15 ppm, product mass tolerance of 50 ppm, default Q-TOF scoring and stringent default “auto validation” settings. For a protein to be considered as identified at least two distinct “validated” peptides were required and an overall summed peptide score for the protein of at least 20. MS/MS spectra were only included with a minimum score of 6 and a scored peak intensity >50%. Modification of cysteine residues by acrylamide was a required modification and oxidation of methionine was allowed as a variable modification. Initially, tryptic cleavage was required and up to two missed cleavages were allowed. In subsequent rounds of searching, semi-tryptic cleavage was allowed for proteins with peptide spectra that had already met the “auto-validation” criteria.

### Lipidomic analysis by liquid chromatography-mass spectrometry of LDs

Lipids from the isolated “fat pads” were resuspended in butanol:methanol (1:1, v/v) to a final concentration of 1 mg mL^−1^. Lipids were chromatographically separated using a Waters BEH C8 column (Waters, Milford, MA) and a binary gradient with a flow rate of 0.2 mL min^−1^ on an Agilent 1290 liquid chromatography (Agilent Technologies). The mobile phases were: A. H_2_O:acetonitrile (10:90, v:v) with 10 mM ammonium formate and 0.2% acetic acid; B. H_2_O:acetonitrile:isopropanol (5:15:80, v:v:v) with 10 mM ammonium formate and 0.2% acetic acid. The gradient was initially held at 1% B for 2 min, then increased to 20% B over the next 3 min, then increased to 70% B over 7 min, followed by a final increase to 90% B over 2 min. The eluted lipids were analyzed on an Agilent 6490 (Agilent Technologies) based on previously published methods (Reynolds et al., 2015). Results were analyzed by integration using Mass Hunter Quantitative Analysis for QQQ, version B.07.01 (Agilent Technologies) followed by export of the data to csv format and analysis in R (R Core Team, 2016).

### Imaging of LDs

LDs isolated from mesocarp and seed tissues were stained with 1 μg mL^−1^ Nile Red (Sigma-Aldrich, CAS No. 7385-67-3). Cytological localization of LDs and other cellular organelles was carried out in *Nicotiana benthamiana* leaves with transient expression of organelle markers. The ER marker is made of the N-terminus signal peptide of AtWAK2 with the ER retention signal His-Asp-Glu-Leu at its C-terminus (Gomord et al., 1997). The first 29 amino acids of baker's yeast (*Saccharomyces cerevisiae*) cytochrome c oxidase IV was used as the mitochondrial marker (Köhler et al., 1997). The peroxisome marker consists of the peroxisome-signal1 (PTS1, Ser-Lys-Leu) fused to the C-terminus of mCherry (Reumann, 2004). The first 49 amino acids of soybean α-1,2- mannosidase I (GmMan1) fused to mCherry was used as the marker for Golgi apparatus (Saint-Jore-Dupas et al., 2006).

*Agrobacterium tumefaciens* strain AGL1 harboring each of the ER, mitochondria, peroxiosome, or Golgi markers fused to mCherry (red) was co-infiltrated with Agrobacterium strains each harboring *P19, DGAT1, WRI1*, respectively, in leaves of 5-week-old *N. benthamiana*, as previously described (Reynolds et al., 2015). Infiltrated tissues were harvested 3 days after infiltration and stained with 1 μM BODIPY493/503 in 50 mM PIPE buffer (Sigma-Aldrich, Cat. No. D3922) immediately prior to observation. Stained tissues were examined on a laser-scanning confocal microscope (Leica SP8) with a water immersion objective. Fluorophore emissions were collected sequentially in double-labeling experiments to avoid bleed-through from broad, overlapping emission spectra. Images were captured as individual planes or as Z-stacks and representative single planes are shown.

### Bioinformatics

Protein sequences were routinely searched using BLAST and aligned using the T-Coffee multiple sequence alignment online system (http://www.tcoffee.org/), and adjusted manually. Hydropathy plots were generated employing the Kyte-Doolittle algorithm, using the ProtScale program at http://www.expasy.ch/tools/protscale.html. The value G in each graph is the grand average of hydropathy (GRAVY) for each protein and was calculated using the GRAVY calculator program at http://www.gravy-calculator.de/.

Multiple-sequence alignments of oleosin amino acid sequence were performed using the ClustalW algorithm of Geneious 9.0 (Kearse et al., 2012) and were manually corrected. Unrooted phylogenetic analysis was carried out using the neighbor-joining method with the Jukes-Cantor model and a phylogenetic tree was displayed using Geneious 9.0.

## Results

### Oil composition of developing *T. sebifera* fruit

Total lipids of seed and mesocarp during fruit development in *T. sebifera* were analyzed by GC, as summarized in Figure 1B. Higher concentrations of total lipids were profiled in seed kernel than mesocarp at early stages. At stage II, for example, seed kernel had accumulated 75% of final lipid content, but the total lipids in mesocarp accounted only for 25% of its total lipids. Despite a slow start, the lipid accumulation in the mesocarp increased more rapidly at the final stages of its development, and reached 57% compared to 41% in the seed kernel by dry weight (DW).

The lipid accumulation during fruit development was accompanied by altered fatty acid composition in both mesocarp and seed kernel tissues. In mesocarp (Figure 1C), an increase in palmitic acid (C16:0) and reduction in linoleic acid (C18:2^Δ9, 12^) were evident as the fruit development progressed. At maturity, the mesocarp tissue featured two major fatty acids, i.e., palmitic acid and oleic acid (C18:1^Δ9^), similar to a typical palm oil (Montoya et al., 2014). In the seed kernel, α-linolenic acid (C18:3^Δ9, 12, 15^) increased quickly during development at the expense of other fatty acids, mostly palmitic acid and linoleic acid, and reached 33% of the total fatty acids at maturity (Figure 1D).

### Isolation of LDs from *T. sebifera* mesocarp and seed kernel tissues

During fruit development, LDs in both mesocarp and seed were observed to increase in both number and size until much of the cytoplasmic space was filled by LDs at the mature stage (stage IV). Immature fruits harvested at stage III were used for LD isolation. At this stage, lipids were actively synthesized and LDs were readily observed in both mesocarp and seed kernel tissues under confocal microscopy following staining with Nile Red (Figure 2A). As outlined in Figure 2B, a “fat pad” containing intact LDs from either mesocarp or seed kernel tissue was isolated and purified using three rounds of Percoll gradient centrifugation. Following staining with Nile Red, the LDs from both tissues were typically spherical under confocal microscopy (Figure 2A).

### Lipidomic analysis of isolated LDs from mesocarp and seed kernels

**Figure 3:**
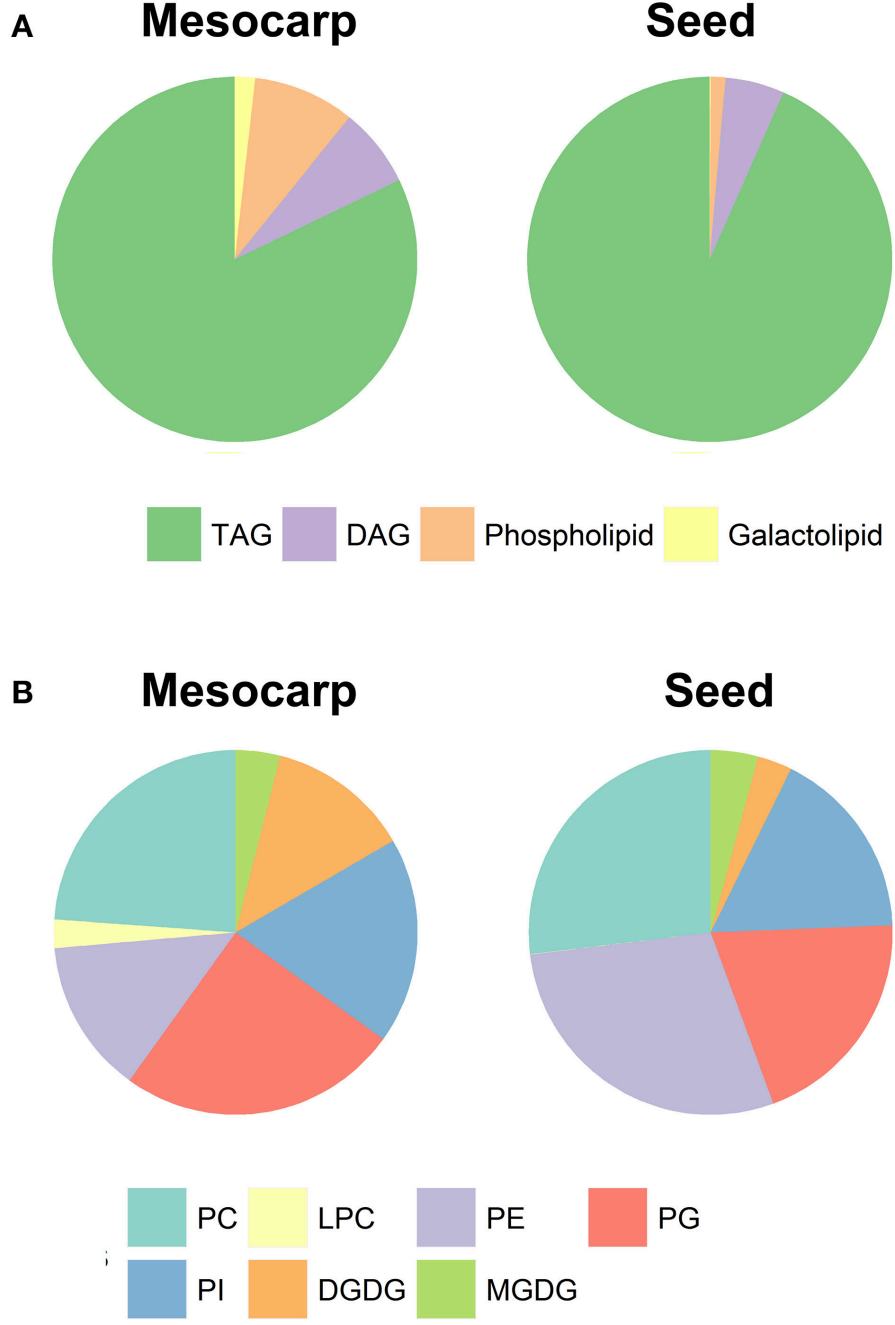
The composition of total **(A)** and polar **(B)** lipids in *Triadica sebifera* LD isolated from stage III immature mesocarp and seed kernel.

The lipidomics approach permitted quantitative analysis of TAG, diacylglycerol (DAG), PL, and galactolipids (GL) species, characterized by their number of carbon atoms and number of double bonds in constituent acyl residues. The classes of non-polar lipids and polar lipids and their relative proportions are summarized in Figure 3A. Not surprisingly, the LDs were highly enriched in TAG, proportion of which was about 8% higher in seed compared to mesocarp. It is interesting to note that the DAG level is relatively consistent between mesocarp and seed kernel LDs.

As shown in Figure 3B, phosphatidylcholine (PC), phosphatidylglycerol (PG), phosphatidylinositol (PI), and phosphatidylethanolamine (PE) were four major PLs in both mesocarp and seed kernel LDs. Although their relative proportions were variable in both mesocarp and seed kernel LDs, none stood out as the dominant type. In contrast to mesocarp, the seed kernel LDs contained very low level of lyso-phosphatidylcholine (Lyso-PC). Among the GLs, monogalactosyldiacylglycerol (MGDG) contents were similar between mesocarp and seed kernel LDs, but much higher level of digalactosyldiacylglycerol (DGDG) was found in mesocarp LD compared to seed kernel LD. Our experimental design did not include phosphatidic acid (PA), phosphatidylserine (PS), cholesterol, or sterol ester (SE) classes due to methodological considerations.

**Figure 4:**
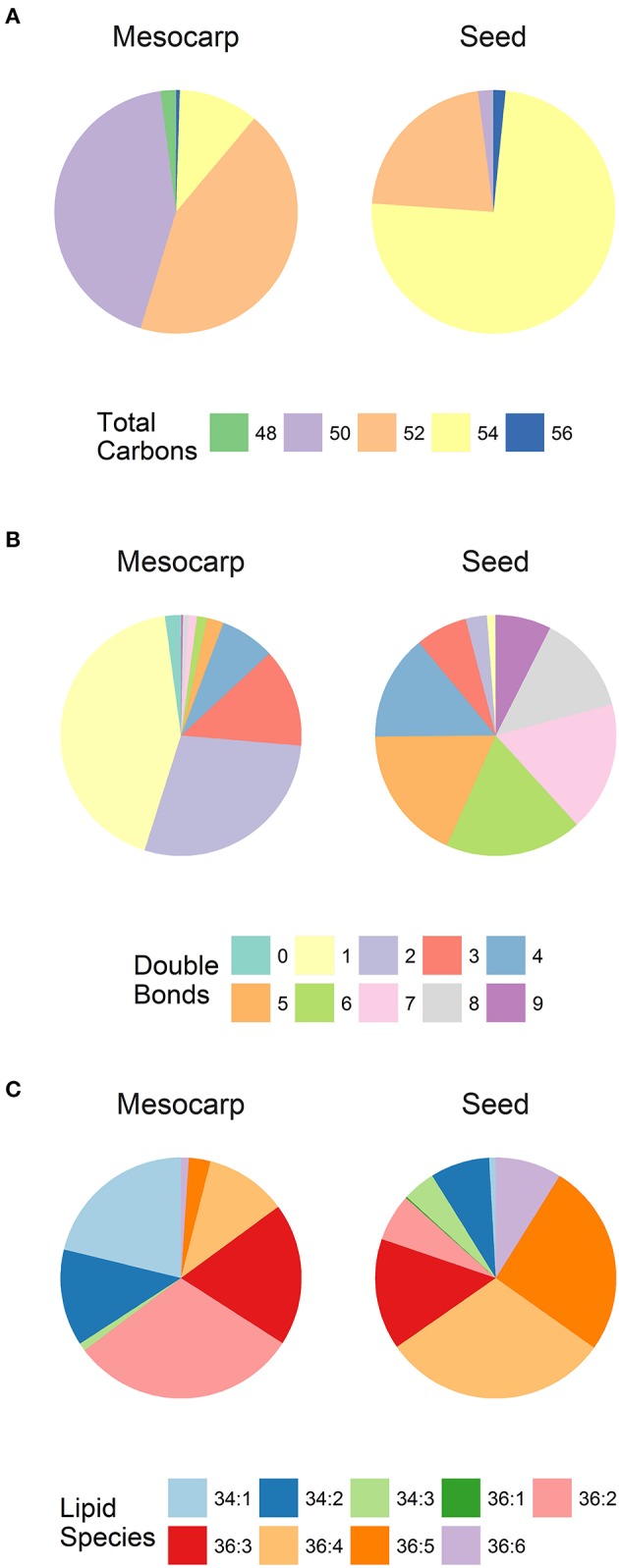
The composition of neutral lipids in *Triadica sebifera* LD isolated from stage III immature mesocarp and seed kernel. **(A)** Total carbon chain length of TAG species; **(B)** Number of double bonds in TAG species; **(C)** DAG lipid species.

The species composition of the two non-polar lipids, TAG and DAG is summarized in Figure 4. As shown in Figure 4A, TAGs in mesocarp LDs were dominated by almost equal proportions of C50 and C52 species which are rich in C16 fatty acids. This is in contrast to seed kernel LDs which contained high level of C54 species containing three C18 fatty acids. The TAG species in mesocarp LDs were dominated by those with high level of saturation (only 1-2 double bonds), in contrast to the seed kernel LDs featured with high level of polyunsaturated TAGs (Figure 4B). A similar trend was observed with DAGs (Figure 4C), where C36:2, C34:1, C36:3, and C34:2 were the major species in mesocarp LD, while the relatively longer chain and polyunsaturated DAGs including C36:4, C36:5, C36:3, and C36:6 were major species in seed kernel LDs.

**Figure 5:**
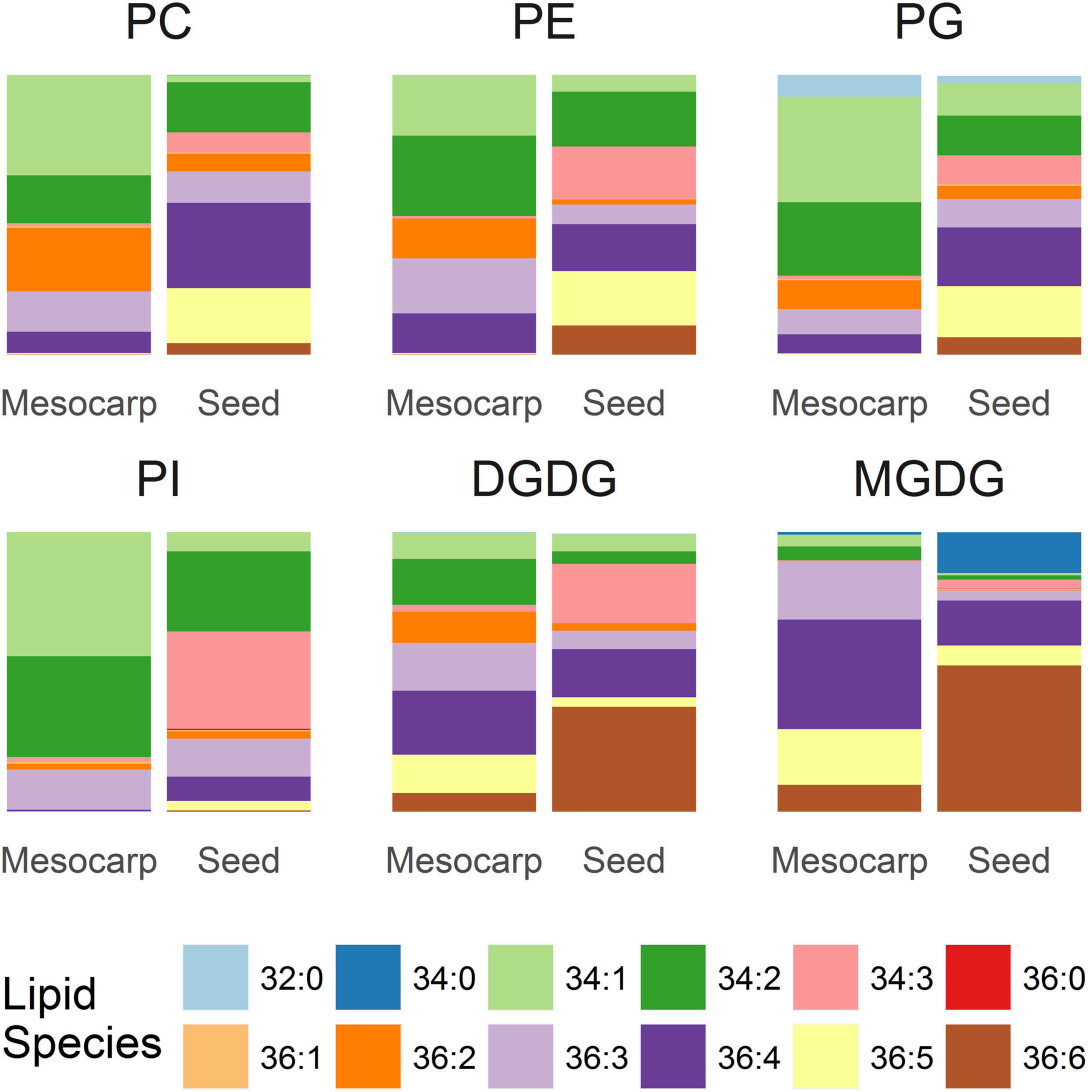
Composition of phospholipids and galactolipids in *Triadica sebifera* LD isolated from stage III immature mesocarp and seed kernel.

The composition of the PLs and GLs is summarized in Figure 5. Among the major PLs in mesocarp, which included PC, PE, PG, the dominant diacyl species were C34:1, C34:2, C36:2, C36:3, and C36:4. The lipid composition of PI was rather simple, consisting of mainly C34:1, C34:2, and C36:3. The proportion of C36 PL species, especially those with multiple double bonds was higher in seed kernel LDs. In seed kernel LDs, C36:4, and C36:5 species were the two major species in PC, PE, and PG, in contrast to PI where C34:2 and C34:3 were the two major species. A fully saturated PL species, C32:0 (C16:0/C16:0) was mainly found in PG from mesocarp LDs. In GL, there was a relatively higher proportion of C36 species, especially those with high levels of unsaturation, relative to PL. C36:6 (di-α-linolenic acid) was the dominant species in both MGDG and DGDG in seed kernels.

### Comparative proteome analysis of LDs sourced from mesocarp and seed kernels

The proteins extracted from “fat pads” were partially fractionated by SDS-PAGE, and processed for in-gel tryptic digestion and LC-MS analysis of the resultant peptides. Two hundred and seven proteins were identified from mesocarp “fat pad” and 54 from seed kernel “fat pad” (Supplementary Data S1, S2). Mass spectral signal intensity does not provide an absolute measure of relative protein abundance. Nonetheless, the total MS signal intensity and numbers of peptides attributed to oleosins and a LDAP protein clearly indicated that these proteins dominated, respectively, in the seed kernel and mesocarp “fat pad” preparations. Other proteins were derived from sequences that are known from the *T. sebifera* transcriptome data (Divi et al., 2016) and annotated with the following functions: lipid metabolism, membrane trafficking, organelle interaction, plant defense, or stress related, cytoskeleton proteins, chaperones, elongation factors, cell signals, storage proteins and proteins with unknown functions (Tables 1, 2).

**Table 1:** Proteins identified (with intensity over E+06) from LDs purified from seed kernel of *Triadica sebifera*.

**Protein name**	**Accession number**	**Spectrum intensity**	**Number spectra**	**Number peps unique**	**Percent coverage**	**BlastP of *A. thaliana***
**LIPID DROPLET ASSOCIATED PROTEIN**						
Oleosin	CL14999.Contig2_sarcocarp	7.77E+08	84	12	40	AT3G01570.1
Oleosin	CL14903.Contig2_embryos	2.65E+08	34	8	94.13	AT3G27660.1
Oleosin	CL11683.Contig1_embryos	3.09E+08	23	5	30.1	AT4G25140.1
Minus strand caleosin	Unigene27688_sarcocarp	2.74E+08	40	8	46.2	AT4G26740.1
Oleosin	Unigene18353_sarcocarp	1.70E+07	15	5	26.9	AT3G18570.1
Oleosin	Unigene11368_All	6.79E+06	10	6	16.8	AT2G25890.1
Steroid binding protein	CL6189.Contig1_sarcocarp	4.61E+06	7	2	12.8	AT3G48890.1
Corticosteroid 11-beta-dehydrogenase (steroleosin)	CL10360.Contig1_All	3.68E+07	25	13	40.6	AT4G10020.1
**LIPID METABOLISM**						
Annexin	CL12939.Contig1_sarcocarp	1.15E+07	16	9	28	AT5G12380.1
Long-chain-fatty-acid CoA ligase	CL2943.Contig1_spornioderm	7.35E+06	4	4	6	AT1G77590.1
Dolichyl-diphosphooligosaccharide-protein	CL6682.Contig2_All	1.42E+06	4	3	5.3	AT4G21150.3
Strictosidine synthase	CL18201.Contig1_spornioderm	1.34E+06	6	4	12.7	AT1G08470.1
**TRAFFICKING**						
SecY protein transport family protein	CL10172.Contig1_All	2.48E+06	4	3	6.9	AT2G34250.2
**ER**						
Calnexin	CL4299.Contig1_All	4.88E+06	11	7	14.7	AT5G61790.1
Cytochrome P450	CL7675.Contig3_All	1.79E+06	3	3	6.8	AT5G42650.1
**MITOCHONDRIA**						
Mitochondrial ATP synthase beta-subunit	CL2076.Contig1_spornioderm	3.35E+06	7	5	13.5	AT5G08670.1
Minus strand ATP synthase F1 subunit	CL15524.Contig1_All	2.27E+06	7	5	10.8	ATMG01190.1
**SIGNAL**						
Bcr-associated protein (14-3-3)	CL6545.Contig2_spornioderm	1.15E+06	6	4	21.5	AT5G42570.1
**STRESS**						
Aspartic proteinase	CL11637.Contig1_All	1.11E+07	14	7	15.1	AT1G11910.1
**CYTOSKELETON**						
Actin1	CL642.Contig3_All	7.96E+07	28	13	45.2	AT5G09810.1
**CHAPERONE**						
Heat-shock protein	CL384.Contig1_embryos	1.92E+07	12	7	35.6	AT5G59720.1
BAG family molecular chaperone	CL3582.Contig2_All	5.95E+06	10	4	12.4	AT5G62390.1
**SEED STORAGE PROTEIN**						
Legumin-like protein	CL2074.Contig1_All	2.74E+08	86	26	54.5	AT5G44120.3
7S Globulin	CL5517.Contig1_All	2.28E+08	69	23	47.9	AT3G22640.1
Late embryogenesis abundant domain-containing protein	CL15240.Contig1_sarcocarp	1.90E+07	29	18	54.4	AT2G42560.1
Late embryogenesis abundant	CL162.Contig1_embryos	1.49E+06	9	6	25.3	AT2G36640.1
Seed storage protein	Unigene25801_sarcocarp	5.77E+07	15	5	67.8	AT5G44120.1
**OTHERS**						
Nutrient reservoir	CL12253.Contig1_embryos	1.22E+07	18	11	22.1	AT2G28490.1
Conserved hypothetical protein	CL2299.Contig3_sarcocarp	6.89E+06	18	11	34.5	AT1G65090.3
Uro-adherence factor A precursor(Uafa)	CL15400.Contig2_spornioderm	6.81E+06	15	7	11.5	AT2G32240.1

**Table 2:** Proteins identified (with intensity over E+06) from LDs purified from mesocarp of *Triadica sebifera*.

**Protein name**	**Accession number**	**Spectrum intensity**	**Number spectra**	**Number peps unique**	**Percent coverage**	**BlastP of *A. thaliana***
**LIPID DROPLET ASSOCIATED PROTEIN**						
LDAP	CL4872.Contig1_All	1.50E+09	224	20	78.2	AT1G67360.1
MLP-like	Unigene14364_All	2.27E+08	110	13	79.3	AT2G01520.1
**LIPID METABOLISM**						
Alcohol dehydrogenase	CL1355.Contig3_spornioderm	1.75E+07	26	10	30.8	AT1G77120.1
Plastid-lipid-associated protein	CL11109.Contig1_spornioderm	8.44E+06	17	9	32.6	AT4G04020.1
Glucose-6-phosphate/phosphate Translocator 2	CL17402.Contig1_spornioderm	7.22E+06	6	2	5.5	AT1G61800.1
Steroid binding protein	CL639.Contig2_All	6.44E+06	18	4	28.7	AT3G48890.1
Lipoxygenase	CL9285.Contig2_sarcocarp	3.48E+06	12	9	21.5	AT3G45140.1
Glyceraldehyde-3-phosphate dehydrogenase	CL9538.Contig1_embryos	2.90E+06	5	4	22.5	AT1G13440.1
Lipase	CL191.Contig9_All	1.55E+06	6	4	9.3	AT3G14360.1
1-Aminocyclopropane-1-carboxylate	CL1964.Contig2_All	1.30E+06	6	3	6.2	AT1G05010.1
**TRAFFICKING**						
PPase	CL1416.Contig1_spornioderm	3.01E+07	37	10	11.5	AT1G15690.1
Pyrophosphate-energized membrane proton pump	CL11743.Contig2_spornioderm	1.68E+07	10	3	4.1	AT1G16780.1
GTP-binding protein	CL17439.Contig1_spornioderm	1.62E+07	30	9	31.1	AT1G28200.1
Ras-related small GTP-binding family protein	CL3891.Contig1_All	9.42E+06	25	11	49.5	AT5G59840.1
GTP-binding protein Sar1	CL1650.Contig1_embryos	8.83E+06	21	6	44	AT4G02080.1
Plastid hexose transporter (sugar transporter)	CL9091.Contig1_spornioderm	6.34E+06	10	4	9.6	AT5G16150.3
B-Cell receptor-associated 31-Like	CL6545.Contig2_spornioderm	6.13E+06	18	7	37.7	AT5G42570.1
Ras 5	CL1909.Contig2_All	5.53E+06	9	6	29.5	AT1G02130.1
Clathrin heavy chain	CL12020.Contig3_All	3.84E+06	18	11	10.4	AT3G11130.1
Rab GTPase	CL2329.Contig1_spornioderm	3.21E+06	11	3	18.4	AT3G18820.1
Ubiquinol-cytochrome C reductase iron-sulfur subunit	CL687.Contig1_embryos	2.87E+06	9	4	19.3	AT5G13440.1
Voltage-dependent anion-selective channel	CL11320.Contig1_spornioderm	2.04E+06	5	4	15.2	AT5G67500.1
Coatomer	CL3781.Contig2_All	2.00E+06	8	7	7.9	AT4G34450.1
AAA-Type ATPase family protein	CL6610.Contig1_All	1.77E+06	10	9	17.2	AT4G04910.1
SecY protein transport family protein	CL10172.Contig1_All	1.24E+06	7	3	5.4	AT2G34250.2
Vesicle-associated membrane protein	CL514.Contig2_sarcocarp	1.08E+06	8	2	10.4	AT2G32670.1
**FATTY ACID SYNTHESIS**						
Acetyl-CoA carboxylase	CL332.Contig1_All	2.43E+07	43	12	32.7	AT5G35360.1
Long-chain Acyl-CoA synthetase	CL13900.Contig1_spornioderm	8.21E+06	28	14	18.9	AT4G23850.1
Ketoacyl-ACP synthase	CL6128.Contig2_spornioderm	7.64E+06	18	8	35.7	AT5G46290.1
Transmembrane proteins 14C	CL3491.Contig1_sarcocarp	3.66E+06	15	3	13	AT2G38550.1
Cytochrome P450 (polyunsaturated fatty acids and eicosanoids)	CL16397.Contig1_All	2.85E+06	11	8	22.8	AT2G45550.1
S-adenosyl-L-methionine synthetase 2	CL441.Contig1_embryos	2.73E+06	7	3	8.6	AT4G01850.2
Very-long-chain enoyl-CoA reductase	CL11516.Contig1_spornioderm	1.92E+06	7	2	7.2	AT3G55360.1
Cytochrome C1	CL7446.Contig1_sarcocarp	1.85E+06	8	3	16.4	AT5G40810.1
Long-chain-fatty-acid CoA ligase	Unigene12451_All	1.75E+06	6	3	5.6	AT1G77590.1
**ER**						
Calnexin	CL4299.Contig1_All	4.98E+06	19	8	19.6	AT5G61790.1
Ribophorin Ii (Rpn2) family protein	CL6682.Contig2_All	4.24E+06	17	8	14.5	AT4G21150.3
Cytochrome B5 isoform 1	CL17079.Contig2_All	2.40E+06	8	4	32.3	AT2G32720.1
7-Dehydrocholesterol reductase	Unigene30429_sarcocarp	2.82E+06	13	2	7.6	AT1G50430.1
Ribophorin I	CL12504.Contig2_All	1.01E+06	3	3	8.5	AT1G76400.1
Translocon-associated protein	CL1956.Contig1_sarcocarp	6.08E+06	4	2	21.3	AT5G14030.4
**MITOCHONDRIA**						
ATP synthase F1 subunit (mitochondrion)	CL15524.Contig1_All	4.80E+07	56	22	42.8	ATMG01190.1
Mitochondrial ATP synthase beta-subunit	CL5099.Contig3_All	8.26E+07	83	18	45	AT5G08680.1
ADP/ATP carrier 2	CL4497.Contig5_spornioderm	3.46E+07	39	11	27.9	AT5G13490.2
Mitochondrial phosphate carrier protein	CL2830.Contig1_All	1.05E+07	27	8	24.7	AT5G14040.1
Mitochondrial processing peptidase beta subunit	CL2813.Contig3_All	9.04E+06	29	14	25.9	AT3G02090.1
ATP synthase CF1 alpha subunit	CL14103.Contig1_sarcocarp	5.53E+06	14	8	19.7	ATCG00120.1
ADP	CL4497.Contig3_spornioderm	4.57E+06	8	2	17.4	AT3G08580.2
Autoinhibited H^+^ ATPase	Unigene6295_All	4.33E+06	17	11	11.2	AT2G24520.1
Prohibitin	Unigene30574_sarcocarp	3.94E+06	12	8	33.1	AT4G28510.1
Aminoadipic semi-aldehyde synthase	CL5935.Contig1_All	3.73E+06	4	3	6	AT4G33150.2
Delta subunit of mitochondria ATP synthase	CL9096.Contig1_sarcocarp	3.16E+06	5	3	13.7	AT5G13450.1
ATPase 10	CL10944.Contig2_spornioderm	3.13E+06	14	9	23.9	AT5G62670.1
Formate dehydrogenase	CL11529.Contig1_All	3.04E+06	16	6	13.8	AT5G14780.1
Prohibitin	CL12038.Contig1_spornioderm	2.44E+06	11	5	21.5	AT5G40770.1
Mitochondrial processing peptidase alpha subunit	CL3048.Contig2_All	2.25E+06	15	6	15.2	AT1G51980.1
Isocitrate dehydrogenase	Unigene26512_spornioderm	1.10E+06	5	2	8.2	AT5G03290.1
Beta-cyanoalanine synthase	CL99.Contig2_All	1.03E+06	6	4	12.4	AT3G61440.1
**SIGNAL**						
ADP-ribosylation factor (Ras superfamily)	CL1130.Contig7_All	4.49E+07	38	8	41.8	AT1G10630.1
Phosphoglycerate kinase	CL13903.Contig1_All	2.67E+07	21	5	59.3	AT1G79550.2
14-3-3 protein	CL17617.Contig2_All	7.51E+06	20	9	31.4	AT3G02520.1
Rab	CL11814.Contig1_embryos	7.17E+06	22	7	36.2	AT1G09630.1
Pyruvate kinase family protein	CL3052.Contig2_All	5.61E+06	12	6	14.5	AT5G08570.1
14-3-3f protein	CL14398.Contig2_All	2.11E+06	14	6	38.3	AT5G65430.2
Transducin family protein/WD-40 repeat family protein	CL5753.Contig3_All	1.78E+06	9	4	4.7	AT3G63460.1
**STRESS**						
Phosphoprotein Ecpp44	Unigene4187_spornioderm	1.62E+08	93	19	61.7	AT1G76180.2
Allene oxide synthase	CL8097.Contig1_spornioderm	5.68E+07	75	18	43.7	AT5G42650.1
AWPM-19-like family protein (tolerance of freezing)	CL12623.Contig1_embryos	6.37E+06	12	4	21.5	AT1G04560.1
Aquaporin	CL13140.Contig1_embryos	4.37E+06	13	2	6.8	AT2G36830.1
Glutathione peroxidase	CL3619.Contig1_spornioderm	4.35E+06	10	4	20.8	AT2G48150.1
Peroxiredoxin	Unigene2500_All	3.75E+06	9	4	27.1	AT1G65980.1
Dead box ATP-dependent RNA helicase	CL2613.Contig2_All	3.33E+06	13	6	18.6	AT3G13920.1
Oxidoreductase, zinc-binding dehydrogenase family	CL8863.Contig1_All	2.55E+06	8	5	21.8	AT4G13010.1
Catalase Cat2	CL4873.Contig2_All	1.64E+06	9	5	11.1	AT4G35090.1
Peroxidase precursor	CL2384.Contig3_All	1.49E+06	7	4	13.6	AT1G71695.1
Glutathione transferase	Unigene1886_spornioderm	1.07E+06	5	3	12.1	AT4G19880.1
**CYTOSKELETON**						
Actin1	CL642.Contig3_All	3.17E+08	114	20	60	AT5G09810.1
Histone H2B1	CL1216.Contig1_embryos	6.43E+07	36	8	47.5	AT5G59910.1
Tubulin beta-3 chain-like	CL4465.Contig14_All	5.42E+07	51	11	35.3	AT4G20890.1
Myosin11	CL15400.Contig2_spornioderm	9.46E+06	42	20	34.8	AT2G32240.1
**PROTEIN METABOLISM**						
Cysteine protease	CL859.Contig1_spornioderm	1.88E+07	18	4	11.1	AT5G43060.1
Glutathione S-transferase	CL8053.Contig1_spornioderm	1.80E+07	21	11	51.2	AT5G41210.1
26S proteasenon-ATPase Subunit 9	CL15761.Contig1_All	3.12E+06	11	5	17.1	AT1G29150.1
Aspartic proteinase precursor	CL11637.Contig4_All	2.83E+06	7	4	12.2	AT1G11910.1
Regulatory particle triple-A 1A	Unigene17101_All	2.48E+06	15	6	15.8	AT1G53750.1
**CHAPERONE**						
Hsp90	CL2416.Contig3_sarcocarp	4.66E+07	60	22	30	AT5G56000.1
Heat shock protein	CL18720.Contig1_spornioderm	2.19E+07	48	17	36.4	AT3G12580.1
Bag family molecular chaperone	CL3582.Contig2_All	4.20E+06	6	2	6.4	AT5G62390.1
Chaperone protein dnaJ	CL16917.Contig1_spornioderm	1.42E+06	5	4	8.8	AT4G39150.2
**ELONGATION FACTOR**						
Elongation factor 1-alpha	Unigene245_All	3.59E+08	109	16	39.8	AT5G60390.3
Ribosomal protein S5/Elongation factor G/Iii/V	CL6208.Contig1_spornioderm	1.28E+07	43	24	32	AT1G56070.1
Elongation factor 1-gamma-like	CL12414.Contig2_All	1.49E+06	7	4	13.1	AT1G57720.2
**OTHERS**						
VIRB2-interacting protein 1	CL3461.Contig1_spornioderm	5.45E+07	26	8	21.7	AT4G23630.1
LURP-one-relate	CL7760.Contig1_All	1.30E+07	11	6	28.2	AT5G01750.2
Carotenoid cleavage dioxygenase	CL2032.Contig1_spornioderm	1.16E+07	21	11	25	AT3G63520.1
GroES-like zinc-binding	CL1724.Contig5_All	5.74E+06	13	8	28.6	AT3G19450.1
Methylenetetrahydrofolate dehydrogenase	CL4451.Contig1_spornioderm	5.60E+06	16	9	38.5	AT3G12290.1
Protein Z	CL13896.Contig1_spornioderm	4.87E+06	20	9	29.5	AT1G47710.1
Mannose-1-phosphate guanyltransferase	CL11530.Contig1_spornioderm	4.74E+06	14	6	18.3	AT1G74910.2
Strictosidine Synthase	CL18201.Contig1_spornioderm	4.72E+06	15	8	22.5	AT1G08470.1
Serine hydroxymethyltransferase	CL11832.Contig2_All	3.87E+06	16	6	19.9	AT4G13930.1
RmlC-like cupins superfamily	CL2510.Contig2_spornioderm	3.40E+06	9	4	42.8	AT5G44120.1
60S Ribosomal protein L9	CL1060.Contig1_sarcocarp	3.13E+06	9	3	19.5	AT4G10450.1
Methylenetetrahydrofolate reductase	CL389.Contig1_spornioderm	2.70E+06	5	2	4.6	AT2G44160.1
Nucleic acid binding protein	CL12290.Contig2_embryos	2.31E+06	12	5	26.2	AT5G16840.2
Endomembrane protein 70 protein family	CL3557.Contig3_spornioderm	1.98E+06	7	4	16.8	AT1G14670.1
Melibiase family protein (A-Galatosidase)	CL8204.Contig2_All	1.96E+06	6	2	6.3	AT3G56310.1
Adenine phosphoribosyl transferase 3 (Nuleotide synthesis)	Unigene22613_spornioderm	1.88E+06	4	3	32	AT4G22570.1
Dihydrolipoamide dehydrogenase	CL6720.Contig2_sarcocarp	1.85E+06	9	6	23.6	AT1G48030.2
Phosphoglucomutase	CL15207.Contig2_All	1.52E+06	14	8	20.3	AT1G23190.1
Nucleolar protein Nop56	CL1859.Contig2_spornioderm	1.43E+06	3	3	8.5	AT5G27120.1
Dolichyl-diphosphooligosaccharide-protein	CL5041.Contig1_All	1.15E+06	8	3	8.5	AT5G66680.1
O-Fucosyltransferase family protein	CL7155.Contig2_sarcocarp	1.12E+06	5	2	8.5	AT1G51630.1
T-Complex protein subunit theta-like	Unigene12720_All	1.05E+06	9	4	10.2	AT3G03960.1
Male sterility protein	CL7994.Contig1_All	1.01E+06	3	3	9.5	AT4G33790.1

**Figure 6:**
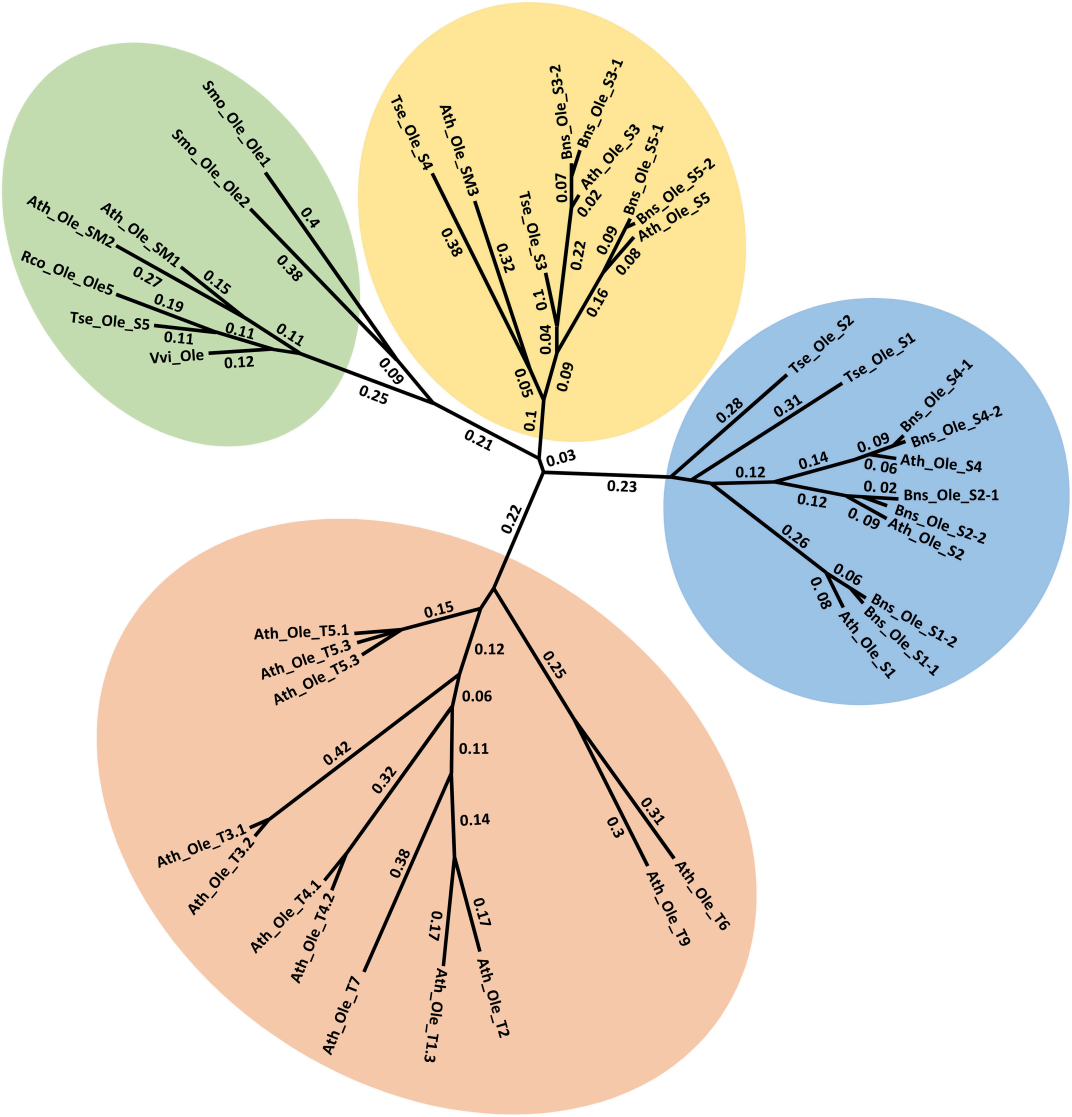
Unrooted Phylogenetic tree of Oleosins derived from *Triadica sebifera* and other plant species. The four previously recognized lineages of Universal (U), seed high (H), seed low (L), and Tapetum (T) are color coded. For presentation clarity, oleosins are labeled with its plant species origin, which include Ath (*Arabidopsis thaliana*), Tse (*T. sebiferum*), Rco (*Ricinus communis*), Smo (*Selaginella moellendorffii*), Vvi (*Vitis vinifera*), Bsn (*Brassica napus*). The phylogenetic tree was constructed using Neighbor Joining method with Jukes-Camtor Models of Geneious 9.0. Branch labels show substitutions per site. The GenBank accession numbers are shown as follows: Ath_Ole_S1 (NP_186806), Ath_Ole_S2 (NP_189403), Ath_Ole_S3 (NP_194244), Ath_Ole_S4 (NP_198858), Ath_Ole_S5 (NP_199934), Ath_Ole_SM1 (NP_175329), Ath_Ole_SM2 (NP_188487), Ath_Ole_SM3 (NP_180160), Ath_Ole_T1.1 (NP_001078542), Ath_Ole_T1.2 (NP_196368), Ath_Ole_T1.3 (NP_001031849), Ath_Ole_T2 (NP_196369), Ath_Ole_T3.1 (NP_196370), Ath_Ole_T3.1 (NP_001119185), Ath_Ole_T4.1 (NP_196371), Ath_Ole_T4.2 (NP_850788), Ath_Ole_T5.1 (NP_196372), Ath_Ole_T5.2 (NP_001078543), Ath_Ole_T5.3 (NP_001119186), Ath_Ole_T6 (NP_196373), Ath_Ole_T7 (NP_196377), Ath_Ole_T8 (NP_196369), Ath_Ole_T9 (NP_001119187); Bns_Ole_S1-1 (ACG69504), Bns_Ole_S1-2 (ACG69505), Bns_Ole_S2-1 (ACG69503), Bns_Ole_S2-2 (ACG69506), Bns_Ole_S3-1 (ACG69513), Bns_Ole_S3-2 (ACG69514), Bns_Ole_S4-1 (ACG69507), Bns_Ole_S4-2 (ACG69508), Bns_Ole_S5-1 (ACG69511), Bns_Ole_S5-2 (ACG69512), Rco_Ole_Ole5 (XP_002511984); Vvi_Ole (XP_002273242); Smo_Ole_Ole1 (EFJ36766), Smo_Ole_Ole2 (EFJ27139). All the *OLEOSIN* gene sequences of *T. sebiferum* were retrieved from public transcriptome database (Divi et al., 2016), which include Tse_Ole_S1 (CL14999.Contig2_sarcocarp), Tse_Ole_S2 (CL14903. Contig2_embryos), Tse_Ole_S3 (CL11683. Contig1_embryos), Tse_Ole_S4 (Unigene11368_All), Tse_Ole_S5 (Unigene18353_sarcocarp).

**Figure 7:**
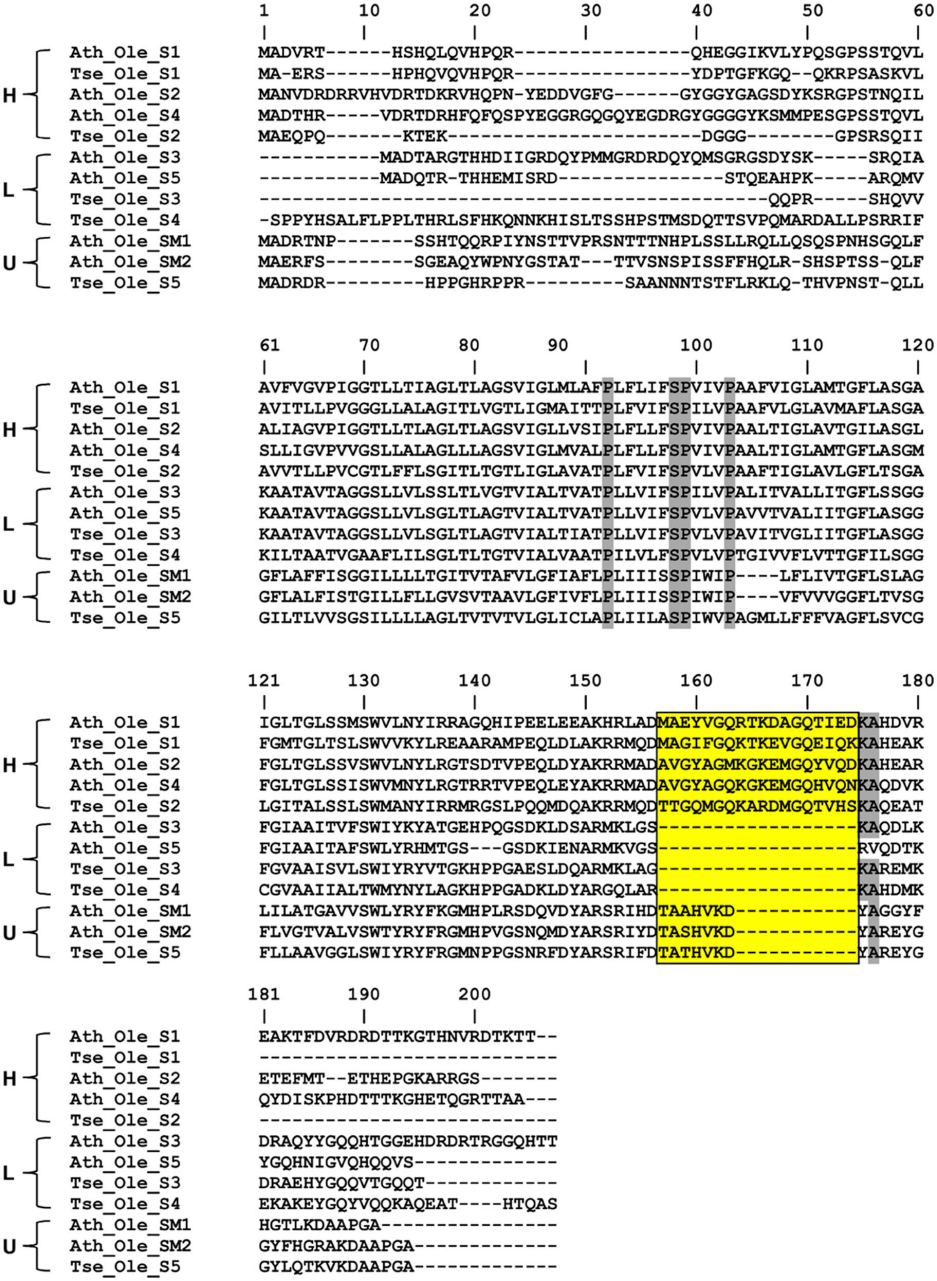
Deduced amino acid sequence comparison of three oleosin isoforms (H, L, and U) derived from diverse plant species. The alignment was generated with ClustW program and then adjusted manually to optimize the alignment. Residues with highest conservation are highlighted with shade. The stretch of four invariable residues of the proline knot (PX_5_SPX_3_P) was also highlighted with shade while the insertion sites in the C- terminus of the H- Oleosin and U- Oleosin, relative to L- Oleosin, are boxed. Oleosins of the selected plant species are indicated with genus name, Ath (*Arabidopsis thaliana*), Tse (*Triadica sebiferum*).

Oleosins, which were previously reported as the major seed LD proteins in seeds of other species, were found to be the most abundant proteins in *T. sebifera* seed LDs (Table 1). Five different oleosins were identified from three categories: H- oleosin (Tse_Ole_S1 and Tse_Ole_S2), L- oleosin (Tse_Ole_S3 and Tse_Ole_S4), and U- oleosin (Tse_Ole_S5) (Figure 6). The previously reported T- oleosin that is tapetum-specific was not found. An alignment of the amino acid residue sequences shows that all the five oleosins contain the hallmark hairpin with a loop of PX_5_SPX_3_P that is highly conserved among plant oleosins (Figure 7). The L- oleosins, including Tse_Ole_S3 and Tse_Ole_S4, are the shortest, and relative to these there is an insertion of seven residues in Tse_Ole_S5, and an insertion of 18 residues in Tse_Ole_S1 and Tse_Ole_S2 in the C- terminus (Figure 7).

Caleosin was the second most abundant protein, behind oleosin, in *T. sebifera* seed kernel LD, but it is absent in the mesocarp LD (Tables 1, 2). Caleosin has been known as a major LD integral protein in oilseeds, which contains a hydrophobic core of about 30 amino acids and a calcium-binding motif in its N- terminus (Katavic et al., 2006; Jolivet et al., 2009; Popluechai et al., 2011).

While LDAP showed the greatest mass spectral signal intensity of all the proteins found in *T. sebifera* mesocarp, another similar protein, major-latex protein (MLP), was also one of the most apparently abundant proteins from mesocarp LD (Table 2). LDAP proteins have previously been found in mesocarp from *P. americana* (Horn et al., 2013) and *E. guineensis* (Loei et al., 2013), but MLP had not been previously observed.

**Figure 8:**
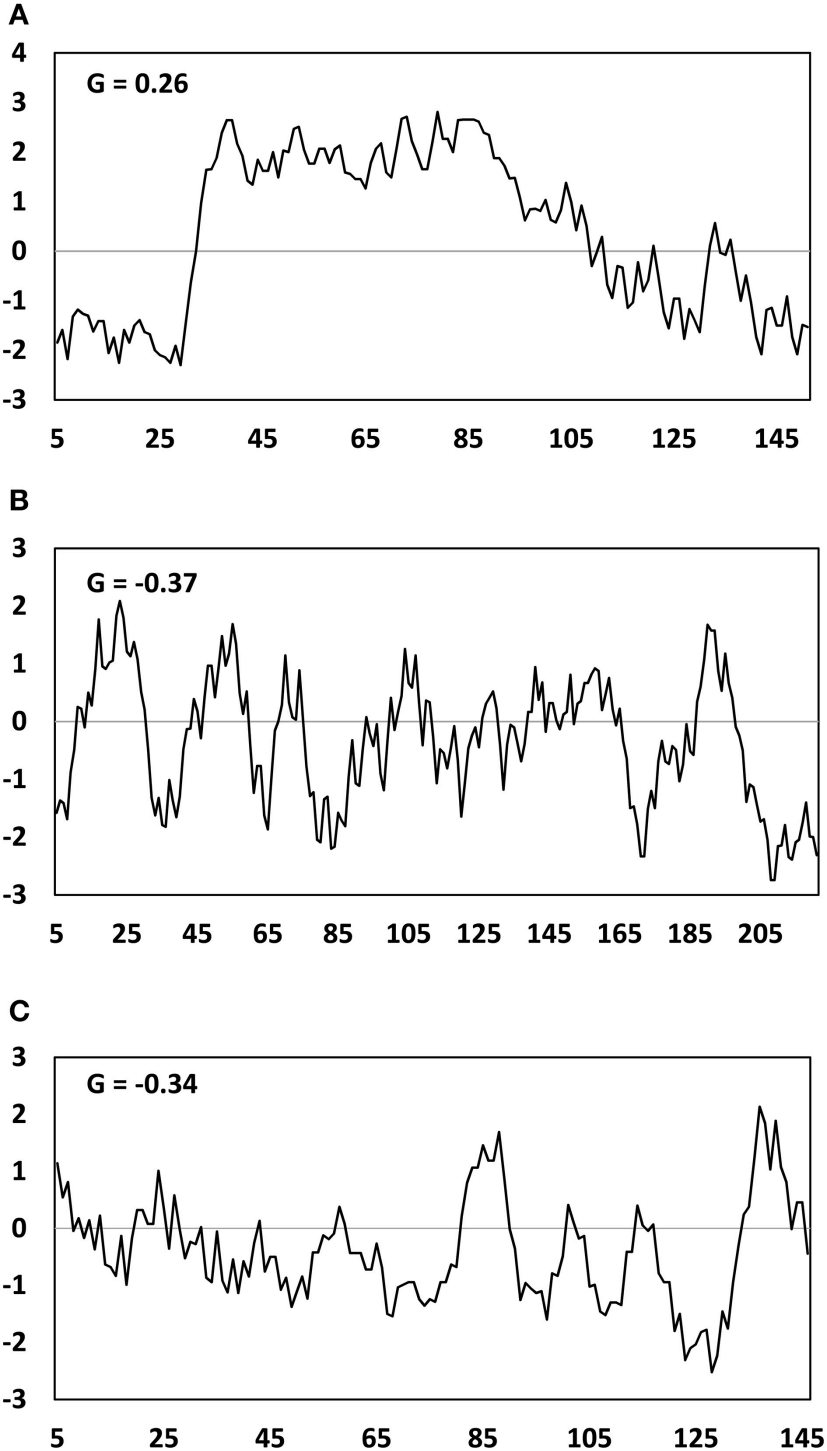
Hydropathy plot of Oleosin **(A)**, LDAP **(B)**, MLP-like **(C)** deduced amino acid sequences from the *Triadica sebifera* proteomic analysis. G represents the GRAVY- grand average of hydropathy value.

GRAVY indices and Kyte Doolittle hydropathy plots were generated for the two major mesocarp LD proteins (LDAP and MLP), along with the major seed kernel LD protein oleosin (Figure 8). The GRAVY index of these three proteins could be ranked from highest to lowest in the order of LDAP (−0.37), MLP (−0.34), and oleosin (0.26), indicating that LDAP and MLP are much less hydrophobic than oleosin. This is consistent with the fact that LDAP and MLP do not contain the hydrophobic core that features in oleosin. However, due to the lack of uniformity in hydrophilicity, it is difficult to determine whether LDAP and MLP are inserted into or merely associated with the mono-layer of PL that coats the mesocarp LDs in *T. sebifera*.

Corticosteroid-11-beta-dehydrogenase which is also known as steroleosin and commonly found in oilseed LDs (Jolivet et al., 2004; Katavic et al., 2006; Liu H. et al., 2015) was identified in *T. sebifera* seeds. In mesocarp LD, a low level of keto acyl synthase that is a component of plastidial *de novo* fatty acid biosynthesis was identified in the mesocarp LD proteome. This is in addition to the identification of a number of other enzymes involved in the fatty acid biosynthesis such as acetyl-CoA carboxylase (ACCase) BC subunit, and long-chain acyl-Coenzyme A synthetases (LACSs) in this tissue. A number of lipases, such as long-chain fatty acid-CoA lipase, were also found in both the seed and mesocarp LDs.

Numerous proteins related to signal transduction and chaperone-like activity, such as AnnAt8, aspartic proteinases, Bcl-2-associated athanogene (BAG) and aquaporin, were identified mostly in the mesocarp LDs. A number of late embryogenesis abundant (LEA) proteins were identified in seed kernel LDs. A variety of proteins that are involved in the regulation of membrane trafficking were also identified (Tables 1, 2). These include a number of small GTPases that regulate vesicle formation and targeting, motor proteins such as myosin that move vesicles on the cytoskeleton, and vesicular trafficking proteins such as Sar1 that regulate vesicle budding and ADP-ribosylation factor 1 (ARF1) that carries out multiple roles in plant cell membrane trafficking (Matheson et al., 2007).

### Subcellular localization of LDs in relation to other major cellular organelles

**Figure 9:**
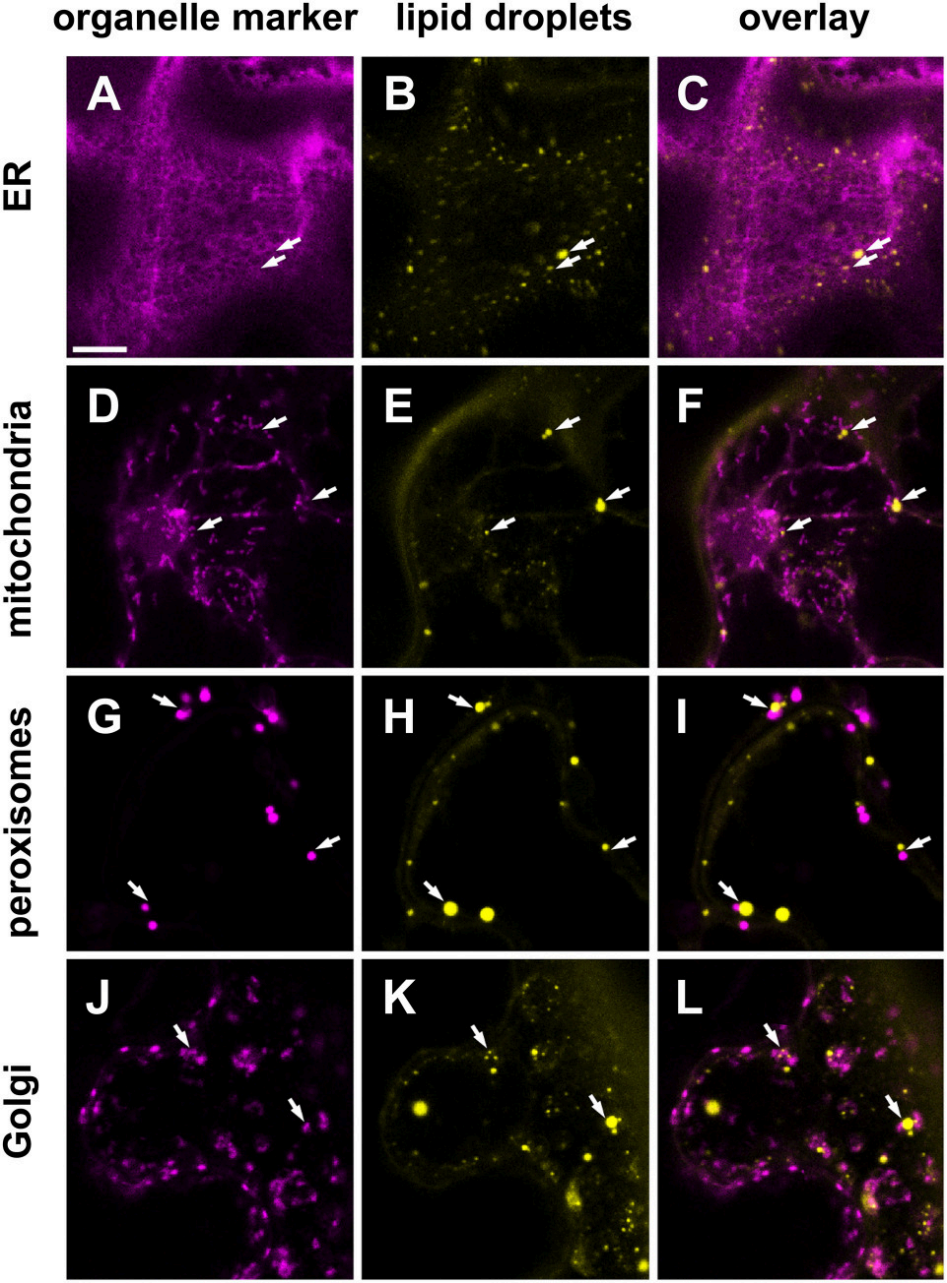
Subcellular localization of LDs (yellow) in *Nicotiana benthamiana* leaves transiently expressing various organelle markers (magenta). Markers for the endoplasmic reticulum (ER, **A–C**) mitochondria **(D–F)**, peroxiosomes **(G–I)**, or the Golgi apparatus **(J–L)**, fused to mCherry were co-infiltrated with *P19, DGAT1, WRI1* in the leaves of 5 weeks old *N. benthamiana*. Arrows highlight examples of LDs found in the vicinity of marked cellular organelles. Scale bar in **(A)** = 10 μm.

To ascertain potential subcellular interactions between LDs and organelles, we transiently expressed different organelle markers by infiltration in *N. benthamiana* leaves and stained these tissues with BODIPY493/503, a fluorescent dye commonly used to stain LDs in living cells. As shown in Figures 9A–C, the LDs were found in the cytoplasm in the neighborhood of the ER network. Some LDs were also observed in the vicinity of mitochondria (Figures 9D–F), peroxisomes (Figures 9G–I), and the Golgi apparatus (Figures 9J–L). These associations are suggestive of subcellular interactions, but were not investigated in detail here.

## Discussion

Although traditionally regarded as a simple repository for stored carbon reserves, emerging evidence suggests that LDs function as a dynamic organelle with multiple functional roles in cellular lipid metabolism, membrane trafficking, and cell signaling (van der Schoot et al., 2011; Chapman et al., 2012; Laibach et al., 2015). We isolated LDs of *T. sebifera* and report the compositions of lipids and proteins of LD purified from both immature seed kernel and mesocarp tissues at a developmental stage with active lipid biosynthesis activities based on lipid biochemistry data. It is understandable that LD structure and composition may differ throughout the fruit development. Nevertheless, this work represents a snapshot of the comparative lipidomics and proteomics of LDs between seed kernel and mesocarp, -the two oleaginous tissues in *T. sebifera* fruit. It warrants further research aiming for a better understanding of the LD dynamics and temporospatial regulations of lipid metabolism.

The LDs have a lower density than aqueous buffer, and hence can be separated from other cellular fractions using centrifugation. In this study, ultracentrifugation was used to isolate LDs that were subsequently used for proteomic and lipidomic analyses. In this work, we have employed Percoll gradient centrifugation which has demonstrated its effectiveness in isolating green algae LDs (Huang et al., 2013). Despite this technical advance, it is still possible that the isolated LD fraction may have been accompanied by a variety of adventitious subcellular components, such as hydrophobic proteins and other lipid-soluble compounds that were not genuinely associated with LDs *in situ*. Thus, to obtain more accurate LD lipidomes and proteomes, the isolated LDs may need further purification using more complicated means such as flow cytometric sorting based on size or surface markers. Nevertheless, the purification methods used in this study are comparable to the plant LD analysis in literature. In this study, some of the identified proteins are typically abundant in any cell type while others are known to be abundant in the tissues used. For example, seed storage proteins such as legumin were observed in the current *T. sebifera* seed LD proteome, which is congruent with previous studies in *H. annuus* (Millichip et al., 1996), *B. napus* (Katavic et al., 2006), and *J. curcas* (Jolivet et al., 2013). The ultrastructure of *J. curcas* seeds revealed by transmission electron microscopy clearly showed that the LDs were tightly associated with protein storage vacuoles (PSVs) (Jolivet et al., 2013). Membranes of the hydrophobic PSVs may adhere to LDs, which makes it very difficult or even impossible to isolate LDs without contamination with seed storage proteins even after stringent washing with salt and urea (Millichip et al., 1996; Katavic et al., 2006).

Lipidome data obtained by LC-MS analysis illustrate a detailed profiling of non-polar lipids and polar lipid species present in LDs derived from *T. sebifera* seed kernel and mesocarp. In mesocarp LDs, the most abundant species of TAG were palmitate-rich C50:1, while α-linoleneate-rich C54-TAGs were dominant in seed kernel LD. Similarly, PC, PE, and PG in seed kernel occur as several polyunsaturate-rich species such as C36:4 and C36:5, in contrast to those with shorter carbon chains and lower levels of unsaturation, such as C34:1 and C34:2, in mesocarp LD. Considering the similar profiles between non-polar lipids and PL in LD, it is tempting to suggest that LD in association with ER may facilitate the inter-conversion of PL and TAG and function as a supply depot for PL biosynthesis as well as a recovery organelle for storage of non-polar lipids (Bartz et al., 2007). Similar lipid profiles were also detected in TAG and DAG. This may reflect that DAG species can be the precursors for TAGs or *vice versa*. Relative to seed kernel, much higher levels of Lyso-PC were detected in mesocarp LD, suggesting a highly active PL recycling therein.

LDs are decorated with membrane integral or loosely associated proteins (Tzen and Huang, 1992; Chapman et al., 2012; Laibach et al., 2015). Each type of organism contains a specific set of key LD-associated proteins, for example, perilipins in mammals (Kimmel et al., 2010), major lipid droplet protein (MLDP) in algae (Moellering and Benning, 2010), and phasins in bacteria (Wältermann and Steinbüchel, 2005). The most abundant proteins in seed LDs are oleosins that are structural low-molecular-mass amphipathic proteins (Huang, 1996; Frandsen et al., 2001; Huang et al., 2009). Oleosins display a conserved architecture with an exceptionally long central hydrophobic region, enabling its stable anchoring in the TAG core of the LDs, flanked by two terminal hydrophilic regions. In the center of this hydrophobic stretch lies a conserved motif containing three Pro residues that are crucial for LD-targeting (Abell et al., 2004; Huang et al., 2013).

Oleosins play a key role in TAG accumulation by controlling the sizes of LDs through the processes of steric hindrance and electrostatic repulsion, which prevent individual LDs from coalescing and fusing (Tzen and Huang, 1992; Siloto et al., 2008). They also play a crucial role in maintaining the stability of seed LDs which can withstand extreme desiccation, rehydration and temperature variation before the TAG stored within is mobilized during seed germination (Rosnitschek and Theimer, 1980). Oleosins are not known to contain any catalytic domains, but a recent study has also suggested that they have bifunctional activities as monoacylglycerol acyltransferase (MGAT) and phospholipase, in addition to their structural role (Parthibane et al., 2012).

Seventeen oleosin genes have been identified in *A. thaliana*, of which five are exclusively expressed in seeds (Siloto et al., 2008). Consistent with the phylogenetic classification in *A. thaliana* (Tzen et al., 1990; Huang and Huang, 2016), five different oleosins belonging to U, L, and H phylogenic lineages were identified in the *T. sebifera* LD proteome. H- oleosin was clearly the most dominant form in *T. sebifera* seed LDs. In developing seeds of sesame (*Sesamum indicum*) (Peng and Tzen, 1998) and *J. curcas* (Popluechai et al., 2011), the H-oleosin accumulates later than L- oleosin, but became the most abundant protein in LDs when seeds approach maturity, suggesting their different roles during lipid accumulation in seeds. Further research is needed to examine whether Tse_Ole_S3 and Tse_Ole_S4 are superior integral LD proteins to Tse_Ole_S1 and Tse_Ole_S2, and could be used for genetic manipulation of plant lipid accumulation, considering previous *in vitro* analysis of LD assembly demonstrated that L- oleosin is more stable than H- oleosin (Tai et al., 2002).

Similar to previous LD proteomic analyses in plants such as *A. thaliana* (Jolivet et al., 2004), *B. napus* (Katavic et al., 2006; Jolivet et al., 2009), *Z. mays* (Tnani et al., 2011), and *J. curcas* (Popluechai et al., 2011), caleosin was also found to be a major protein in *T. sebifera* seed kernel LDs, but at a much lower abundance than oleosin. It is generally believed that caleosin may anchor to LDs in a manner similar to oleosin, hence playing an important role in lipid accumulation as well as LD degradation during germination (Poxleitner et al., 2006). In addition to a structural role, caleosin was also shown to have peroxygenase activity and be involved in plant oxylipin biosynthesis and defense responses (Partridge and Murphy, 2009).

Steroleosin, a membrane-associated NADP+-binding sterol dehydrogenase, also known as corticosteroid 11-beta-dehydrogenase, has also been identified in the seed LD proteome, based on its sequence homology with sesame steroleosin Sop2 (Lin et al., 2002). Sterol-binding proteins were also identified in both seed and mesocarp LDs, but they appeared to be more divergent from a typical steroleosin.

Unlike seed, the predominant protein found in *T. sebifera* mesocarp LDs was LDAP, rather than oleosin. The LDAP protein shares homology with small rubber particle protein (SRPP), which was named for its association with rubber particles storing rubber (cis-1,4-polyisoprene) in laticifer cells of a number of plants, such as rubber tree (*Hevea brasiliencsis*) (Wititsuwannakul et al., 2008; Berthelot et al., 2014), and Russian dandelion (*Taraxacum brevicorniculatum*) (Collins-Silva et al., 2012; Hillebrand et al., 2012). It does not share any detectable primary or secondary structural features with oleosin, nor does it harbor any transmembrane domains. Three *SRPP* homologs, *SRP1, SRP2*, and *SRP3*, have been identified and characterized in *A. thaliana* (Kim et al., 2016). Horn et al. (2013) reported LDAP1 and LDAP2 as the dominant protein in the *P. americana* LD proteome, both of which are homologs of *A. thaliana* SRP3. RNA-Seq data from five stages of developing *P. americana* mesocarp indicated that the transcript levels of these two *LDAPs* were highest during the middle maturity stage of fruit development when oil accumulation peaked. Similarly, the expression of an ortholog of the *P. americana LDAPs* was substantially higher in the mesocarp of *E. guineensis* than date palm (*Phoenix dactylifera*), which stores very little oil (Bourgis et al., 2011). Unlike oleosin, SRP/LDAP may not be integral LD proteins. However, it remains unknown at present how the LDAP proteins assemble on the LD surface, despite speculation that they may become associated with the LD periphery in an isotropic manner (Sookmark et al., 2002).

The *T. sebifera* seed LD proteome also featured late embryogenesis abundant proteins (LEAs) that are normally found at high levels during the late embryogenesis stage of seeds and in vegetative organs, especially under stress conditions such as cold, drought, or high salinity (Ingram and Bartels, 1996). It has been proposed that LEAs may help to protect against numerous abiotic stresses by controlling the proper folding and conformation of both structural and catalytic proteins (Hasanuzzaman et al., 2013). LEA was also found in abundance in other seed LD proteomes such as *J. curcas* (Liu H. et al., 2015) and *H. annuus* (Thakur and Bhatla, 2015). It is possible that LEAs interact with LD membranes and reduce dehydration-induced damage, prevent LD coagulation and maintain LD integrity by acting as chaperone to prevent the aggregation and/or inactivation of proteins under dehydration during seed maturity. Annexin was also identified only in the seed LD. Its orthologous gene in *A. thaliana, AnnAt8* (At5g12380), was shown to exhibit hundreds-fold increases in transcription levels under dehydration and salt stress (Yadav et al., 2016). Its overexpression in Arabidopsis and tobacco plants resulted in higher rates of seed germination, better plant growth, and higher chlorophyll retention than wild type plants under abiotic stress treatments (Yadav et al., 2016).

Compared with their mammalian, microbial, or algal counterparts, LDs purified from *T. sebifera* seed and mesocarp tissues seem to lack many key enzymes involved in lipid biosynthesis. For example, we did not identify diacylglycerol acyltransferase (DGAT) which has been shown to associate with *C. elegans* LDs at the ER interface (Xu et al., 2012). However, such a low abundance of lipid biosynthesis-related proteins in *T. sebifera* LDs is consistent with previous proteomics studies in *A. thaliana* (Jolivet et al., 2004), *B. napus* (Jolivet et al., 2004), and *J. carcus* (Liu H. et al., 2015).

A number of genes involved in tolerance to environmental stresses have also been identified in *T. sebifera* seed and mesocarp LDs, such as calnexin, aspartic proteinases, and BAG. Calnexin is a highly conserved ER chaperone protein that participates in protein folding *via* calcium-binding (Liu D. Y. et al., 2015). Plant aspartic proteinases have been implicated in protein processing and/or degradation, senescence, stress responses, and programmed cell death (Simões and Faro, 2004). The BAG family is an evolutionarily conserved group of co-chaperones that modulate numerous cellular processes (Williams et al., 2010). It was recently demonstrated that the interaction between BAG6 and aspartic protease is necessary for triggering autophagy, providing a key link between fungal recognition and the induction of cell death and resistance in plants (Li et al., 2016).

One of the major distinctions between the seed kernel and mesocarp tissues in *T. sebifera* is that a large number of organelle-related and membrane trafficking-associated proteins were co-purified with mesocarp LD. This may reflect the *in vivo* interactions of mesocarp LDs with ER, mitochondria, peroxisomes, protein storage vacuoles, small Golgi vesicles, the cytoskeleton, and the plasma membrane. In *T. sebifera* mesocarp LDs, a considerable number of proteins potentially involved in vesicle trafficking and transport were identified, including RAB, RAS-related GTP-binding family proteins, coatomer α, β, and γ subunits. ADP-ribosylation factor 1 (ARF1) was also observed, and is known to carry out multiple roles in plant cell membrane trafficking through spatially regulated recruitment of coatomer and elements of the Golgi matrix (Matheson et al., 2007). The presence of proteins involved in vesicular transport such as GTPases supports the hypothesis that LDs are dynamic organelles that may play a role in neutral lipid transport between cellular organelles. LD movements along microtubules have been demonstrated in live-cell imaging of the *Drosophila* embryo (Welte et al., 1998) and mammalian HuH-7 cells (Targett-Adams et al., 2003). However, this remains to be observed in a plant system.

It is worthy of note that a number of enzymes involved in fatty acid biosynthesis were identified in mesocarp LD. The presence of plastidial proteins does not necessarily correspond to contamination. Instead, it may reflect a genuine association between LD and plastidial membranes. The close proximity of the ER to plastid envelop membranes has been widely reported in algae and lower plants (reviewed by Wang and Benning, 2012). The continuity of the ER and the outer envelope of chloroplasts (McLean et al., 1988), and LD enrichment at the ER-to-chloroplast contact sites (Wang and Benning, 2012), suggests a possible role of these contact sites in lipid metabolism. In higher plants, the association of LDs with developing chloroplasts was also observed in embryogenic pea leaves (Kaneko and Keegstra, 1996). The significant presence of MGDG and DGDG that are characteristic of plastidial lipids in LD lipidome indicates LD-plastidial membrane association. The relatively higher proportion of these galactolipids in mesocarp LD compared to seed kernel LD may also reflect the different extent of plastidial association between these two types of LDs as suggested in proteomic analysis.

In this study, we have illustrated that LDs were often found in close proximity to a number of organelles including ER, mitochondria, peroxisomes, and Golgi. LDs originate from the ER, which is considered as the main site of TAG biosynthesis and where the LD biogenesis machinery resides (Ohlrogge and Jaworski, 1997). LD formation seems to occur at specific membrane microdomains in the ER where non-polar lipids accumulate until the LD reaches a critical size to bud off and form an independent organelle (Wältermann and Steinbüchel, 2005; Chapman et al., 2012). A recent study suggested Seipin, an ER membrane protein implicated in LD biogenesis (Gomord et al., 1997), is critical for the nascent ER-LD contacts and facilitates the incorporation of lipids and proteins into the growing LD in human cells (Salo et al., 2016). The separation of LDs from the ER is likely controlled by the GTP/GDP state of Rab18, which regulates the interaction between the two organelles (Martin et al., 2005).

The presence of LD proteins predicted to be associated with mitochondria is also consistent with other reported LD proteomic studies on mammalian adipocytes, liver, and lactating cells (reviewed by Zehmer et al., 2009). LDs have also been found close to mitochondria in transgenic potato tubers where TAG levels were boosted about 100-fold compared to wild type (Liu et al., 2017). In plants, it has been established that there are two parallel pathways of β-oxidation carried out in mitochondria and the peroxisome (Wood et al., 1992; Masterson and Wood, 2001). The peroxisome maintains a continuous β- oxidation activity that could be essential in removing harmful free fatty acids, e.g., those produced by protein and lipid turnover. In contrast, β- oxidation in mitochondria switches on during times of intense biosynthetic activity at the critical stages of plant development, supplying the acetyl CoA and/or ATP needed for the biosynthesis of membrane and acyl lipids in response to lipid metabolism and development requirements.

## Conclusion

The comparative proteomics and lipidomics analyses of *T. sebifera* seed and mesocarp LDs in this study are in agreement with other recent studies suggesting that LDs could be actively engaged in lipid metabolism, lipid storage, membrane trafficking, protein degradation, and cellular signaling through interactions with various cellular organelles. Future studies need to focus on identifying the molecular machinery that mediates protein-protein interactions, and the physiological functions of multiple LD proteins identified in the *T. sebifera* proteome. This will be especially useful for the exploration of utilizing non-seed high-biomass plant tissues that are normally discarded when seeds are harvested for food, which could be used as sources of TAGs for food oils and biodiesel, and prevent competition between food and energy uses for crops.

## Author contributions

YZ, MT, PC performed proteomics analysis. YZ, PS, AE performed lipidomics analysis. YZ, VR, RW performed microscopy analysis. QL, RW, WC, SS, JP, and TV conceived the idea and designed the experiments. All authors were involved in the data analysis and preparation of manuscript.

### Conflict of interest statement

The authors declare that the research was conducted in the absence of any commercial or financial relationships that could be construed as a potential conflict of interest.
